# Is FDG Metabolic Tumor Volume in lymphoma really happening?

**DOI:** 10.2967/jnumed.123.267022

**Published:** 2024-02-22

**Authors:** Sally F Barrington, Anne-Ségolène Cottereau, Josée M. Zijlstra

**Affiliations:** 1 King’s College London and Guy’s and St Thomas’ PET Centre, School of Biomedical Engineering and Imaging Sciences, King’s College London, UK; 2 Department of Nuclear Medicine, Cochin Hospital, APHP, Paris Cité University; 3 Amsterdam UMC, Department of Hematology, Vrije Universiteit Amsterdam, Cancer Center Amsterdam, Netherlands

**Keywords:** lymphoma, metabolic tumor volume, standardization, methodology, evaluation

Tumor burden influences prognosis in lymphoma, with unidimensional bulk used for risk assessment and decisions about radiotherapy consolidation (1). Total metabolic tumor volume (TMTV) using fluorine-18-fluorodeoxyglucose-positron emission tomography ([^18^F]-FDG-PET) emerged 10 years ago as a promising biomarker (2) that was superior to bulk (3). However, until now MTV has not been used in clinical practice nor trial design.

We attribute this to lack of common methodology, the perception that measurement is difficult and the (un)availability of software tools. Consensus is also required about which ‘tumor’ areas to include (4). Furthermore, MTV has been evaluated in datasets providing binary ‘cut-offs’ to divide patients into prognostic groups, which are data driven and population dependent (5).

## What is changing?

### A new benchmark method has been established

Prognostication and interobserver agreement are equally good irrespective of the measurement method (6), so choice should reflect ease of use. One method has emerged as simple, quick to perform using academic and commercial softwares and which closely matched the visual perception of nuclear medicine reads from six published methods in diffuse large B-cell lymphoma (DLBCL) (7) and Hodgkin Lymphoma (8). The delineation method uses a standardized uptake value (SUV)≥ 4.0 and minimum individual lesion volume of 3mls. The SUV of 4.0 limits physiological uptake that requires editing compared with lower thresholds and reduces underestimation of heterogeneous lesions compared with percentage SUV thresholds. The 41% maximum SUV threshold, although frequently studied, has increased variability across softwares depending on definition of maximum SUV, whether defined as maximum in the TMTV or if lesional, the clustering algorithm used to outline lesions. The minimum volume reduces measurement complexity without significantly influencing TMTV. The SUV4.0 method is insensitive to uptake time, the presence/absence of later progression in patients (7) and least sensitive to reconstruction method, including ultrahigh sensitive reconstructions (9) used in advanced technologies e.g. Total Body PET-CT. In approximately 80% of DLBCL cases, minimal reader interaction was required e.g. removing physiological uptake with single clicks, achievable in 2-3 minutes (7) with additional manual editing in 20%.

The vision for standardization of MTV measurement was outlined in this journal (4) with a proposal for a benchmark dataset using a common method with consensus MTV values and segmentations as outputs. Data from the ‘MTV road map’ have been presented involving 12 readers from 9 countries using 3 academic softwares. Readers analyzed 60 cases from 3 lymphoma subtypes (10). TMTV measurement was unaffected by software used with close reader agreement in 51/60 cases. Disagreement was mainly due to interpretation of diffuse splenic uptake with smaller less clinically relevant differences due to manual editing of physiological uptake and optional inclusion of lesions <3mls.

The benchmark will soon be made publicly available so readers can check the reliability of MTV measurements using local software and their clinical interpretation. New measurement methods including artificial intelligence approaches can be evaluated against the benchmark with both tested in the same dataset, provided patient outcomes are known, to determine whether newer methods improve prognostication, reduce reader time and/or improve agreement. The benchmark can also be used to explore questions such as the prognostic relevance of the spleen.

A concern about the transition to one method could be whether research using other methods might be wasted and that SUV4.0 has not been widely tested in indolent, albeit less common subtypes. A statistical method for combining batches (ComBat) of data using different methods (11) has been successfully applied in retrospective trial datasets (12).

Nonetheless, having a standardized approach for prospective study in aggressive lymphoma is a critical step for universal adoption of MTV as a biomarker.

### Incorporation into new prognostic indices

Other major developments are testing of TMTV in large datasets, expression as a continuous variable and incorporation with established risk factors.

The SUV4.0 method was explored in 1214 patients with newly diagnosed DLBCL (13) by the PETRA consortium in 5 international trials. Firstly, the best statistical relationship was derived to associate MTV with progression-free-(PFS) and overall survival (OS). The relationship was a linear spline with 2 coefficients, such that the same incremental change had different impact on survival above and below the median. MTV performed better than the international prognostic index (IPI) (comprised of binary cut-offs for age, lactate dehydrogenase, stage, performance status (PS) and > 1 extranodal site) (14). Most IPI factors proved redundant when combined with MTV. The optimal ‘international metabolic prognostic index’ (IMPI) included 3 factors of MTV and age (as continuous variables) and stage (I-IV). Its continuous nature means PFS can be predicted for individual patients by entering MTV, age and stage in a simple excel spreadsheet (https://petralymphoma.org/impi). IMPI allows for intelligent trial design, selecting a PFS cut-off where the benefit of a novel treatment will likely outweigh the risk of standard treatment in high-risk patients. The integration of MTV with patient factors has also been explored in 2174 patients from trial and real-world datasets, with PS identified as an independent risk factor, with MTV plus PS outperforming the IPI (12). Optimal selection of high-risk patients is very relevant as new treatments such as CAR-T cell therapy and bispecific monoclonal antibodies are currently being tested in Phase-III trials in first and second-line DLBCL.

### How should we build on the success of MTV?

The success of MTV has generated interest in other radiomic features, which can be measured once TMTV is delineated. Independent prognostic value of disease dissemination was first reported by Cottereau et al (15), e.g. the maximum distance between lesions (Dmax). Biological explanations for this phenomenon were recently explored in Hodgkin lymphoma (16). The PETRA consortium suggested the potential to replace “stage” in IMPI by “Dmaxbulk”, the maximum distance between the bulkiest and the furthest lesion, with a small incremental benefit for a “radiomics” score that also included PS and peak SUV (17).

Preliminary reports that integrate PET with emerging molecular markers in circulating tumor DNA (ctDNA) (18) may further improve baseline and dynamic risk. Methods to establish reliable dynamic MTV measurement are also being explored (19).

Confidence in TMTV is growing with agreement about standardization. A similar pragmatic approach of a simple, albeit not perfect method, led to widespread adoption of the Deauville score for lymphoma (20).

Now MTV needs to feature in trial design, either alone or within prognostic indices like IMPI for risk stratification. MTV (+/- other radiomic features) should be prospectively evaluated at baseline and interim with liquid biomarkers for minimal residual disease to develop clinical decision tools.

The first trial using MTV, Deauville score and ctDNA to risk adapt treatment is already underway in Hodgkin lymphoma (www.clinicaltrials.gov/study/NCT04866654).

In conclusion, MTV for risk stratification in DLBCL is feasible now in the clinic and being evaluated in a clinical trial in Hodgkin lymphoma. A benchmark dataset will be available soon for standardization of measurement by PET centers, software developers and vendors.

The ‘time to prepare for risk adaptation in lymphoma by standardizing measurement of metabolic tumor burden’ is over: it’s time to get on board.
